# Single-shot simultaneous intensity, phase and polarization imaging with metasurface

**DOI:** 10.1093/nsr/nwae418

**Published:** 2024-11-22

**Authors:** Yanjun Bao, Baojun Li

**Affiliations:** Guangdong Provincial Key Laboratory of Nanophotonic Manipulation, Institute of Nanophotonics, College of Physics and Optoelectronic Engineering, Jinan University, Guangzhou 511443, China; Guangdong Provincial Key Laboratory of Nanophotonic Manipulation, Institute of Nanophotonics, College of Physics and Optoelectronic Engineering, Jinan University, Guangzhou 511443, China

**Keywords:** metasurface, single-shot imaging, optical imaging, Jone matrix, intensity, phase and polarization

## Abstract

Optical imaging of the intensity, phase and polarization distributions of optical fields is fundamental to numerous applications. Traditional methods rely on bulky optical components and require multiple measurements. Recently, metasurface-based (MS-based) imaging strategies have emerged as a promising solution to address these challenges. However, they have been primarily limited to capturing partial information of the three parameters, tailored to specific optical fields, which poses challenges when addressing arbitrary field distributions and achieving three-parameter imaging. In this study, we introduce an MS-based approach for single-shot optical imaging that simultaneously captures all three parameters of optical fields with arbitrary intensity, phase and polarization distributions. We experimentally validate the versatility of our method by conducting imaging of various types of optical fields with arbitrary well-defined distributions. The strategy presented in our work is expected to open up promising avenues for diverse applications, including imaging and optical communications.

## Teaser

A metasurface-based approach is proposed for single-shot optical imaging that simultaneously captures all the three parameters of optical fields with arbitrary intensity, phase, and polarization distributions.

## INTRODUCTION

Optical fields are primarily characterized by three parameters: intensity, phase and polarization. The precise imaging of these parameters holds significant importance across a broad spectrum of applications, including microscopy, material characterization and biophotonics. Traditional imaging techniques rely on bulky optical components, such as lenses, waveplates, polarizers and phase retarders, and often require multiple measurements. For instance, lenses can image the intensity in a single shot, while polarization imaging demands several specific rotational combinations of polarizers and waveplates [1]. For phase imaging, techniques such as phase-shifting interferometry [2–5] and the transport of intensity equation (TIE) [6–8] are commonly used but also involve multiple measurements. The former requires a minimum of three phase-shifted reference fields for interference [2]. The TIE method is susceptible to noise and requires multiple measurements across several planes with varying transmission distances [7]. Additionally, the algorithm of TIE may not converge and may result in inaccurate results for specific phase distributions like those with a vortex pattern [7].

Metasurfaces, consisting of 2D planar structures with artificial atoms, have emerged as a pivotal tool for manipulating the intensity, phase and polarization of light, unlocking advancements in a variety of optical applications [9–20]. Their planar configuration, combined with versatile optical control, presents innovative opportunities for device miniaturization and enables single-shot imaging. For instance, metasurface-based (MS-based) metalenses [21–28] can replace traditional lenses for intensity imaging, offering significantly reduced thickness and simplified integration. For polarization imaging, metasurfaces have been utilized for single-shot imaging of all four Stokes components [28,29]. Regarding phase imaging, several methods, such as differential phase imaging (DPI) [30–34] and TIE [35], have been employed with metasurfaces. However, these approaches impose certain constraints on the input fields. The DPI method relies on the gradient of the optical field, necessitating input fields with uniform intensity [30,33] to avoid amplitude differentiation effects or linear polarization and ensure two identical polarization components during interference [30–33]. Moreover, the DPI method only captures the gradient along one direction in a single shot [30,31,33], precluding the complete reconstruction of phase distribution. Recently, a strategy combining metasurfaces with polarization cameras has been developed to enable complex amplitude field imaging [34]. For the TIE method, metasurfaces facilitate simultaneous recording of two images at different propagation distances [35], which also requires 45° linear polarization of the incident field to ensure identical transverse magnetic and transverse electric polarization distributions. Consequently, existing MS-based imaging techniques are restricted to capturing partial optical parameters for specific field distributions. Hence, developing a method capable of imaging arbitrary optical fields and achieving single-shot three-parameter imaging is highly desirable.

In this work, we have demonstrated MS-based single-shot imaging of all three parameters simultaneously for optical fields with arbitrary intensity, phase and polarization distributions. Our work offers three significant advancements compared to previous MS-based imaging strategies: it addresses arbitrary optical fields, captures all three parameters simultaneously, and achieves single-shot three-parameter imaging without multiple measurements of each parameter. This achievement is rooted in the careful design of different components of the Jones matrix of the metasurface, optimized to diffract the orthogonal polarization of the input field and generate the desired reference fields. We have verified the versatility of our method by conducting optical imaging on various types of arbitrary optical fields with well-defined distributions.

## RESULTS AND DISCUSSION

### Design of MS-based single-shot three-parameter imaging

**Figure 1.**
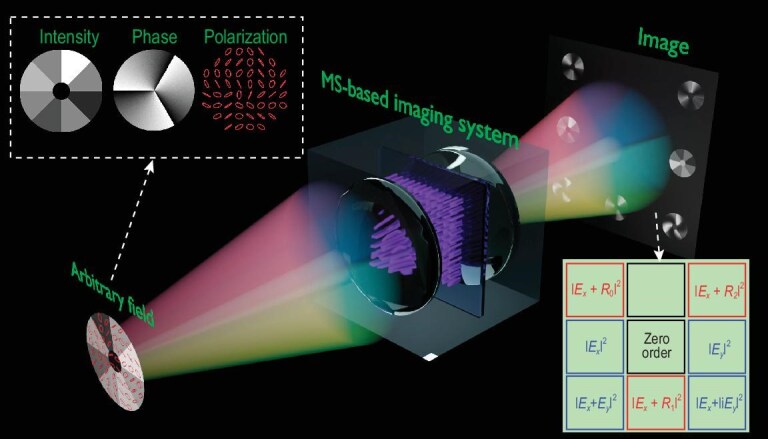
MS-based single-shot three-parameter imaging of an arbitrary optical field. The input field exhibits arbitrary intensity, phase and polarization distributions. The captured image contains seven sub-images at distinct regions. Three sub-images (red rectangles) represent the interference between *E_x_* and reference fields *R_m_*. The other four sub-images (blue rectangles) capture intensities of *E_x_, E_y_, E_x_* + 1i*E_y_* and *E_x_* + *E_y_*.

Figure 1 presents our MS-based optical imaging system, designed for single-shot three-parameter imaging of input fields with arbitrary intensity, phase and polarization distributions. Upon passing through the imaging system, the input field is diffracted to produce seven sub-images located at distinct regions (inset of Fig. 1). Among them, three are intentionally designed to represent the interferences between the *x*-component of the electric field (*E_x_*) and the reference fields (*R*_m_), as highlighted with red rectangles. Here, *R*_m_ represents an optical field with uniform amplitude and constant phase 2m*π*/3, where *m* takes values 0, 1 and 2. The intensities of the three captured sub-images (labeled *I*_0_, *I*_1_ and *I*_2_) allow us to obtain the *E_x_* phase distributions *φ*_x_ as follows [2] (see details in Supplementary Section 1, SMI):


(1) \begin{equation*}
{{\varphi }_x} = {\mathrm{atan2}}( {\sqrt 3 \left( {{{I}_1} - {{I}_2}} \right),2{{I}_0} - {{I}_1} - {{I}_2}}),
\end{equation*}

where the function atan2(*y, x*) returns the phase of a complex number in the form *x* + 1i*y*.

The remaining four sub-images, marked with blue rectangles, are designed to capture the intensities of *E_x_, E_y_, E_x_* + 1i*E_y_* and *E_x_* + *E_y_*, labeled as ${{I^{\prime}}_0}$, ${{I^{\prime}}_1}$, ${{I^{\prime}}_2}$ and ${{I^{\prime}}_3}$, respectively. This procedure is essentially the same as the conventional measurement of the four Stokes polarization parameters [1]. Through these image intensities, we extract not only the intensities of *E_x_* and *E_y_*, but also the phase difference between them, which can be calculated as (see details in SM1):


(2) \begin{equation*}
{{\varphi }_x} - {{\varphi }_y} = {\mathrm{atan2}}\left( {{{{I^{\prime}}}_2} - {{{I^{\prime}}}_0} - {{{I^{\prime}}}_1},{{{I^{\prime}}}_3} - {{{I^{\prime}}}_0} - {{{I^{\prime}}}_1}} \right).
\end{equation*}

In conjunction with (1), we can deduce both the intensity and phase distributions of *E_x_* and *E_y_*, which are equivalent to the intensity, phase and polarization of the field. Therefore, such a system enables single-shot three-parameter imaging of arbitrary input fields.

**Figure 2.**
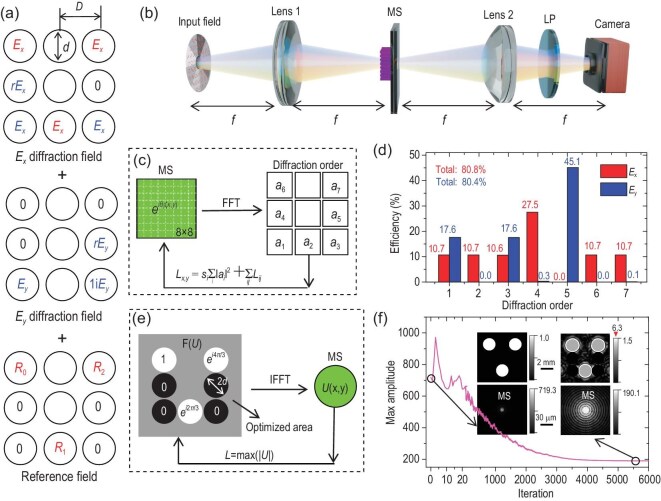
A metasurface design for three-parameter optical imaging. (a) Categorization of captured image fields into *E_x_, E_y_* diffraction fields and the reference field *R*. These fields span across seven distinct regions, with certain regions exhibiting null intensities. Each diffraction order image is confined within a circle of diameter *d*, with a center-to-center spacing *D* between sub-images. The *E_x_* and *E_y_* diffraction fields, located in the mid-left and mid-right regions, are augmented by a factor *r* = 1.6. (b) Schematic of the key optical components: two lenses, a metasurface and a linear polarizer. (c) Depiction of the gradient-based optimization aiming to enhance the efficiency of *E_x_* and *E_y_* fields and reach their respective desired diffraction orders. The metasurface features an 8 × 8 periodic pattern with pure phase, where *a*_i_ represents the seven orders of interest closest to the zeroth order. The period of the metasurface *P* and the center-to-center distance between sub-images *D* are related by the equation *D* = *fλ*/8*P*. (d) Optimized efficiency of the seven orders for both *E_x_* field (red bars) and *E_y_* field (blue bars). (e) Illustration of the optimization of the intensity of the reference field using a gradient-based approach. Gray areas outside the seven circles indicate the optimization regions. The loss function is established as max(|*U*|), with *U* denoting the metasurface distribution. (f) A plot of loss value (max amplitude of metasurface) against iteration number. Insets display the initial and final optimized distributions of F(*U*) (top) and corresponding metasurface distributions (bottom).

The output fields can be categorized into three distinct groups: *E_x_* diffraction field, *E_y_* diffraction field and the reference field *R_i_*, as shown in Fig. 2a. These fields within each category are distributed across discrete regions, with certain regions exhibiting null intensities. The maintenance of zero intensity is of importance to prevent any unwanted cross-interference or noise introduction into other categories. Additionally, precise calibration of the amplitudes and phase across different regions in each category is essential. For instance, the *E_x_* fields in the three regions designed to interfere with reference fields must maintain the same complex-amplitude coefficients to extract the phase correctly. Similarly, the two *E_y_* fields designated for interference with *E_x_* fields should exhibit the same amplitude but a phase difference of *π*/2. The *E_x_* and *E_y_* fields, positioned in the mid-left and mid-right regions, do not interfere with others, which may lead to a lower peak intensity compared to other regions. To ensure that their peak intensities are consistent with other sub-images, the amplitudes of the two fields are augmented by a factor of *r* = 1.6. Detailed considerations regarding the coefficients of each field are provided in SM2.

To realize such an output field, we design an MS-based optical system, including two lenses (Lens 1 and Lens 2 with a focal length *f*), a metasurface and a linear polarizer, as illustrated in Fig. 2b. The two lenses establish a 4*f* imaging system, with the metasurface placed at the central Fourier plane, serving to diffract the incoming optical fields into distinct orders. These orders are subsequently captured by the camera via Lens 2, resulting in an image segmented into seven distinct sub-images. The linear polarizer is set at 45° relative to the x axis, enabling the interference of the fields between *x* and *y* components. Note that these seven diffraction orders have different polarization states and cannot be filtered by the linear polarizer simultaneously.

In the absence of the linear polarizer, the output field generated by the MS-based optical system can be deduced as follows:


(3) \begin{equation*}
E_i^{out}(x,y) = F\left[ {{{U}_i}(\eta ,\nu )} \right] \otimes E_i^{in}( - x, - y).
\end{equation*}

Here, *F* represents the Fourier transform operator and $\otimes$ denotes the convolution operator. The spatial coordinates at the input and output planes are represented by (*x, y*), while (*η, ν*) are the spatial coordinates associated with the *x* and *y* directions at the metasurface plane. $E_i^{in}$, $E_i^{out}$ and ${{U}_i}$ stand for the input field, output field and the complex amplitude of the metasurface, respectively, with *i* indicating either the *x* or *y* component.

### Metasurface design of *E_x_* and *E_y_* diffraction fields

To generate the diffracted orders of *E_x_* and *E_y_* fields, the metasurface can be architected with a periodic pattern, incorporating specific Fourier coefficients. A direct formulation of the metasurface can be expressed as the summation of seven plane waves, represented as $\sum\nolimits_i {{{a}_i}\exp (ik_x^i\eta + ik_y^i\nu )} $. Here, *a*_i_ signifies the complex coefficients of different diffraction orders, with certain coefficients equating to zero for null diffractions (Fig. 2a). The wavevectors along the *x* and *y* directions, $k_x^i$ and $k_y^i$, are determined by the central positions of the seven sub-images (*x_i_, y_i_*) and can be expressed as $k_x^i = {{k}_0}{{x}_i}/f$ and $k_y^i = {{k}_0}{{y}_i}/f$, with *k*_0_ being 2*π*/*λ*. However, a significant limitation of this approach is the diminishing efficiency as the number of plane waves increases. For instance, when considering *E_x_* and *E_y_* fields with six and three diffraction orders respectively, the overall efficiencies are only 17.4% and 35.2% (SM2).

To address this issue, we employ a gradient-based optimization method to design the metasurface (Fig. 2c). The metasurface is designed with an 8 × 8 periodic pattern with pure phase ${{e}^{i{{\theta }_i}}}$, which is subjected to optimization. The Fourier transformation of the metasurface has 8 × 8 diffraction orders, and only the seven closest to the zeroth order are of interest, denoted as *a_i_* (Fig. 2c). In the optimization algorithm, the loss functions *L_x_* and *L_y_* are defined as the summation of the absolute square of *a*_i_ with a prefixed ratio *s_i_*. The ratio *s_i_* equals to -1 for those associated with existing fields and +1 for those with null intensities. Additionally, an additional loss term *L_ij_* is introduced to ensure the consistency of Fourier coefficients across various orders. For example, in loss function *L_x_*, the Fourier coefficients of *a*_2_, *a*_6_ and *a*_7_ are identical. To enforce this, a term of ${{| {1 - {{a}_2}/{{a}_6}} |}^2} + {{| {1 - {{a}_2}/{{a}_7}} |}^2}$ is incorporated into *L_ij_*. More details of the constraints for other Fourier coefficients and the optimization method are provided in SM2. Following this optimized approach, the efficiencies for *E_x_* and *E_y_* fields are significantly enhanced to 80.8% and 80.4% respectively, as illustrated in Fig. 2d.

### Metasurface design of the reference field

Here, we employ the existing optical fields to generate the reference fields. When compared with conventional interferometric phase imaging, which necessitates an additional optical beam from a known laser source as the reference field, our approach presents distinct advantages. It not only simplifies the optical measurement but also proves effective in scenarios where obtaining a reference beam from an unknown source is impractical. The crucial concern here is how to consistently maintain uniform references and null fields within specified regions of interest (bottom panel of Fig. 2a), regardless of variations in the input optical field. Referring to Equation 3, we configure the Fourier transform of the metasurface (*F*(*U*)) to exhibit patterns similar to those of reference fields, but with the diameter of the seven circles augmented to 2*d*, depicted in Fig. 2e. This design, when convoluted with an arbitrary field, generates the desired field distributions within the designated regions (see SM3 for details).

The complex amplitude of the metasurface *U*, can be determined by performing an inverse Fourier transformation on the *F*(*U*) pattern. With a unity magnitude in the three circular regions and zeros elsewhere in the Fourier plane, the maximal amplitude of the metasurface *U* is computed to be 719.3 (inset in Fig. 2f). The distribution of the metasurface with high amplitude values predominantly centers around a narrow central region. However, such straightforward operation can lead to low reference intensity when the metasurface amplitude is normalized, thereby compromising interference contrast. It is notable that only the seven circular regions with diameter *d* contribute to field retrieval. Therefore, in the *F*(*U*) pattern, areas outside these seven circles with diameter 2*d* (gray regions in Fig. 2e) can be optimized to minimize the maximal amplitude of the metasurface, thereby increasing the intensity of the reference field.

We employ a gradient-based algorithm (see SM3 for details), using a loss function of max(|*U|*), as depicted in Fig. 2e. The initial distribution of *F*(*U*) begins with a unity magnitude in the three circular regions and zeroes elsewhere, consistent with the approach previously used. Figure 2f shows the loss value as a function of the iteration which reaches convergence after 6000 iterations. Post-convergence, the peak amplitude of the metasurface is reduced to 190.1 from an initial 719.3, signifying a 14.3-fold enhancement in reference intensity. In this scenario, the fields in the optimized area of *F*(*U*) pattern manifest a varied distribution, and the high-amplitude values of the metasurface pattern are more widely dispersed compared to the initial design (inset of Fig. 2f). We verify the generation of reference fields and find that the intensities and phases can be uniformly manifested within the delineated three circular regions (see SM3). In this case, the interference between the diffracted *E_x_* field and reference field yields a max/min intensity ratio of 5.14, a sufficient contrast for phase extraction (see SM3).

### Metasurface design of the Jones matrix

We proceed to the design of the Jones matrix metasurface, aiming to realize all three functions described above. For the *xx* component of the Jones matrix, we set ${{J}_{xx}} = 0.5{{e}^{i{{\theta }_x}}} + 0.5{{U}_{\mathrm{norm}}}$, where *θ_x_* represents the optimized metasurface phase for *E_x_* diffraction field and *U*_norm_ is the normalized metasurface pattern for reference fields. To ensure coefficient consistency between *E_x_* and *E_y_* fields, the *yy* component is set as ${{J}_{yy}} = 0.39{{e}^{i{{\theta }_y}}}$, with *θ_y_* denoting the optimized metasurface phase for *E_y_* diffraction field. The factor 0.39 is deduced from $0.5/\sqrt {1.645} $, where 1.645 (17.6%/10.7%, Fig. 2d) is the efficiency ratio of the single diffraction order between *E_x_* and *E_y_* fields. Additionally, a reference field term of 0.61*U*_norm_ is incorporated into *J_yy_* to ensure unity peak amplitude. In this design, reference fields are encoded in both *x* and *y* polarizations, and can both interfere with *E_x_* fields, supported by the linear polarizer. For certain cases, the induced intensity of the reference field may be insufficient to achieve a suitable contrast for interference. This challenge can be effectively addressed through several strategies, including adjusting the linear polarizer axis, modifying the field of view size or rotating the metasurface (see SM3).

**Figure 3.**
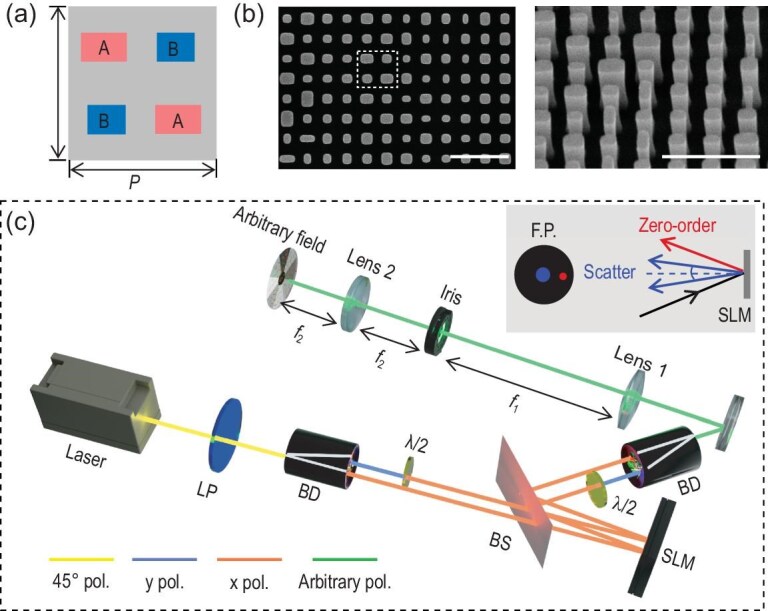
Design of Jones matrix metasurface and optical set-up to generate arbitrary field. (a) Schematic of the tetratomic micropixel unit of the metasurface for constructing the Jones matrix. Each pixel contains two unique rectangular nanoblocks labeled as A and B. (b) Scanning electron microscopy (SEM) images of the fabricated metasurface (partial view). The white rectangle indicates a micropixel with four nanoblocks. Scale bar: 1 μm. (c) Optical set-up to generate an optical field with arbitrary intensity, phase and polarization distribution. The inset details the positions in the Fourier plane (F.P.) of the incident zeroth order and the field scattered by the spatial light modulator (SLM). (LP, linear polarizer; BD, beam displacer; *λ*/2, half waveplate; BS, beam splitter; M, mirror).

We employ a tetratomic micropixel consisting of two distinct rectangle nanoblocks, termed meta-atoms *A* and *B*, for constructing the Jones matrix [36,37] (Fig. 3a). Given that the off-diagonal entries of the Jones matrix are zero, both meta-atoms possess zero rotational angles. The phase shift values for the two meta-atoms along the *x* and *y* axes can be directly deduced from the established Jones matrix. Full wave finite-difference time-domain (FDTD) simulations are performed to create a library detailing the transmission and phase shifts dependent on the nanoblock's transverse dimensions. The desired phase shifts are obtained by appropriately choosing the transverse dimensions of the nanoblocks. The micropixel's period is *P* = 700 nm and is designed for *λ* = 780 nm. More details are provided in SM4.

The metasurface sample, designed with a diameter of 600 μm, is fabricated on a 600 nm-high crystal silicon layer that is transferred on glass substrate. The patterns are then defined by electron beam lithography and a reactive ion etching process. The SEM images, showing both the top and oblique perspectives, are illustrated in Fig. 3b. The details of the fabrication procedure can be found in the ‘Materials and Methods’ section.

### Generation of an arbitrary optical field and experimental verification

Generally, any optical field distribution, characterized by arbitrary intensity, phase and polarization, can be decomposed into two independent optical fields polarized along *x* and *y* directions, each possessing distinct amplitude and phase distributions. Therefore, we can construct such an optical field by modulating its two polarized components separately. The experimental set-up is shown in Fig. 3c. In this set-up, the incident light is initially split into horizontal and vertical polarization components, which then illuminate the left and right halves of the spatial light modulator (SLM) screen. Here, the pure phase pattern in the SLM can be used to encode arbitrary independent amplitude and phase information [38] (see SM5 for details). Subsequently, these two beams are coherently superimposed, generating an arbitrary optical field with predefined intensity, phase and polarization distributions. By illuminating the SLM at a slight oblique angle, the unwanted zeroth order can be shifted away from the Fourier plane's center (red point in the inset of Fig. 3c), which can be easily filtered by the iris. The details of the set-up can be found in the ‘Materials and Methods’ section.

**Figure 4.**
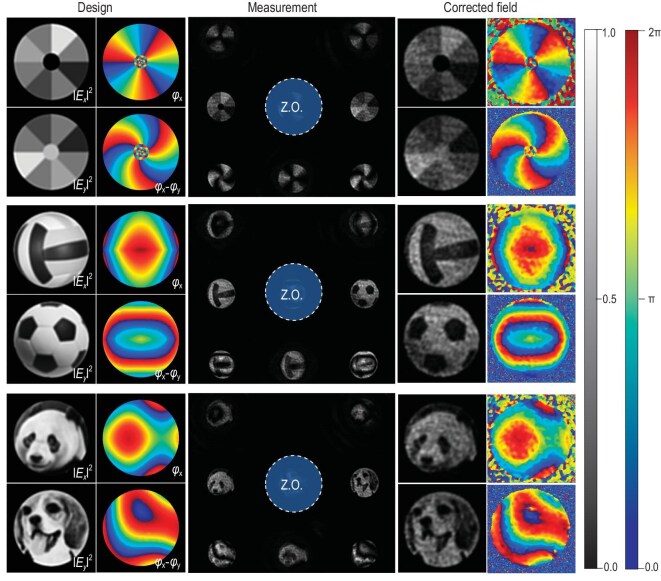
Experimental verification of single-shot three-parameter optical imaging. The leftmost column displays three distinct optical fields, each characterized by unique intensity and phase distributions for the *x*- and *y* components (i.e. ${{| {{{E}_x}} |}^2}$, ${{| {{{E}_y}} |}^2}$, *φ_x_* and *φ_x_*-*φ_y_*). The center column reveals the measurement results. The zero-order (ZO) images are intentionally concealed to enhance visibility. The right column presents the fields retrieved post image correction.

The described optical set-up provides the flexibility to generate an arbitrary optical field with well-defined distribution, which serves as a benchmark to assess the accuracy of our proposed MS-based imaging system. To illustrate this capability, we have designed three general optical fields, as presented in the first column of Fig. 4. These fields exhibit varying intensity and phase distributions in *x*- and *y* components (i.e. ${{| {{{E}_x}} |}^2}$, ${{| {{{E}_y}} |}^2}$, *φ_x_* and *φ_x_*-*φ_y_*), including special phase distributions like vortex patterns that are unfeasible with the TIE method. The results of our measurement, illustrated in the second column of Fig. 4, reveal seven distinct sub-images. The zeroth order image in the center has been manually obscured to avoid interference with observation.

When the incident field is diffracted by the metasurface into several orders, it creates certain angles with the lens's principal axis, leading to imaging aberrations. This is especially apparent in the distortion seen in the four corner sub-images in measurement. To rectify this distortion, an image correction process is employed for all seven sub-images, with specifics outlined in SM6. The corrected field distributions for all three cases are displayed in the third column of Fig. 4, all agreeing well with our original designs. The observed speckles in the measured images primarily arise from the coherence of the laser beam and possibly the inherent limitations of encoding complex-amplitude using SLM, and thus are unrelated to the metasurface design (Fig. S5 in SM5). The efficiency of the metasurface for imaging is addressed in SM7. Since the incident field on the metasurface represents the Fourier transformation of the original incident pattern, the efficiency varies and depends on the original field distribution. The measured efficiency for each of the three types of optical fields ranges from 17.5% to 22.4%, a value nearly acceptable for practical uses.

## CONCLUSIONS

In summary, we have demonstrated an MS-based single-shot imaging system capable of capturing optical fields with arbitrary intensity, phase and polarization distributions. The primary optical set-up employs a 4*f* system with the metasurface placed at the Fourier plane. The system features two lenses with identical focal lengths of 15 mm and a metasurface diameter of 600 μm, resulting in a numerical aperture (NA) of 0.02 and a spatial resolution of ∼19 μm. This spatial resolution can be adjusted by changing the metasurface diameter or the focal length of Lens 1. Additionally, the simultaneous capture of seven diffraction images reduces the field of view (FOV) to ∼1 mm. The FOV can also be modified by altering the focal length of Lens 1. There is always a trade-off between spatial resolution and FOV: increasing the focal length of Lens 1 enlarges the FOV but reduces spatial resolution, and vice versa. The optimal balance between these parameters depends on the specific imaging application requirements.

For the metasurface design, the Jones matrix is carefully designed to generate the desired reference field and diffract the incident *x* and *y*-polarized fields into distinct orders with specific complex-amplitude coefficients. Notably, we have incorporated optimizations into the metasurface design to maximize the efficiency of both the reference field generation and the diffraction of *x* and *y*-polarized light. The design in our work enables the imaging of arbitrary fields with single shot, effectively addressing the limitations inherent in traditional methods. Specifically, it eliminates the need for multiple measurements of the three optical parameters, reduces reliance on a laser source for the reference beam in conventional phase measurements, and overcomes the incapacity of the traditional DPI method to measure phase distributions of arbitrary fields, etc. We believe that our work represents a significant advancement in optical field imaging, offering a highly efficient and versatile solution with broad applicability in areas including microscopy, materials science and optical communications.

## MATERIALS AND METHODS

### Metasurface fabrication

A commercial silicon-on-insulator (SOI) wafer, initially having a device layer thickness of 1200 nm, was initially transferred to a glass substrate through adhesive wafer bonding and deep reactive ion etching (DRIE) techniques [39]. Subsequently, the thickness of the device layer was further reduced to 600 nm by utilizing inductively coupled plasma (ICP) etching. Following this, a 300-nm-thick layer of hydrogen silsesquioxane (HSQ) was spin-coated onto the substrate at a rate of 4000 revolutions per minute and then baked on a hot plate at 90°C for 5 minutes. Next, a 30-nm-thick layer of aluminum was deposited onto the HSQ layer through thermal evaporation to serve as the charge dissipation layer. The metasurface mask on the HSQ layer was created using electron beam lithography at an acceleration voltage of 30 kV. After the exposure, the aluminum layer was removed using a 5% phosphoric acid solution, and the resist was developed with tetramethylammonium hydroxide (TMAH) for 2 minutes at room temperature (25°C). Subsequently, inductively coupled plasma-reactive ion etching (ICP-RIE) was employed to transfer the pattern into the silicon film. Finally, the samples were immersed in a 10% hydrofluoric (HF) acid solution for 15 s to eliminate any residual HSQ mask. The samples were then cleaned with deionized water and dried using nitrogen gas.

### Generation of arbitrary field distributions

The experimental set-up for arbitrary field generation is shown in Fig. 3c. The linear polarization of an input laser beam is oriented at 45° after passing through a linear polarizer. A beam displacer (BD40, Thorlabs) subsequently splits this beam into horizontal and vertical polarization components. The two components, propagating parallelly, illuminate the left and right halves of the SLM (PLUTO-2-NIR-011, Holoeye) screen, where distinct phase patterns are imposed to encode both amplitude and phase information [38]. Since the SLM exclusively responds to *x*-polarization, a half-wave plate is positioned before the SLM to convert the incident *y*-polarization to *x*-polarization. Subsequently, one of the reflected *x*-polarized beams is transformed to *y*-polarization via another half-wave plate. It is superposed coaxially with the remaining *x*-polarized field using a second beam displacer, resulting in a versatile optical field with specified intensity, phase and polarization distributions. The generated field is then directed through a lens pair, forming a 4*f* optical system, with an iris placed at the Fourier plane to filter the zero order of the incident laser. We purposely set the laser to strike the SLM at a slight oblique angle. As a result, the zeroth order shifts away from the Fourier plane's center (highlighted by the red point in the inset of Fig. 3c), enabling easy filtering by the iris. This oblique incidence imposes a gradient phase on the SLM, which can be compensated by the applied phase of the SLM to align the center of the angular spectrum of the generated fields with the center in the Fourier plane (marked by the blue point in the inset of Fig. 3c). The focal lengths of the two lenses (*f*_1_ = 150 mm and *f*_2_ = 50 mm) are selected to ensure that the resultant optical field adopts a circular shape with a diameter smaller than *d* = 1 mm.

## Supplementary Material

nwae418_Supplemental_File
