# Supplementary material for: Single-shot simultaneous intensity, phase and polarization imaging with metasurface
Source: Natl Sci Rev. 2024 Nov 22;12(3):nwae418. doi: 10.1093/nsr/nwae418 (PMC11887858; doi:10.1093/nsr/nwae418)
Supplement: nwae418_Supplemental_File [file nwae418_supplemental_file.docx]

**Supplementary Materials for**

**Single-shot simultaneous intensity, phase, and polarization imaging with metasurface**

Yanjun Bao*, and Baojun Li*

Guangdong Provincial Key Laboratory of Nanophotonic Manipulation, Institute of Nanophotonics, College of Physics & Optoelectronic Engineering, Jinan University, Guangzhou 511443, China

*Corresponding Authors: Y. Bao ([yanjunbao@jnu.edu.cn](mailto:yanjunbao@jnu.edu.cn)), B. Li (baojunli@jnu.edu.cn)

**Table of Contents**

**Section 1. Parameter retrieval with seven sub-images**

**Section 2. Multi-order diffraction design with gradient-based optimization**

**Section 3. Optimization of reference field**

**Section 4. Jones matrix metasurface design with nanoblock elements**

**Section 5. Encoding arbitrary complex-amplitude distribution with pure-phase SLM**

**Section 6. Lens choose and image correction**

**Section 7. Metasurface efficiency measurement**

**Section 1.** **Parameter retrieval with seven sub-images**

In this section, we present the derivation of intensity, phase, and polarization distributions based on the captured seven sub-images. We assume that the incident fields are represented as follows: for *x*-polarization, , and for y-polarization, . Thus, the measurement of intensity, phase, and polarization is equivalent to the measurement of full field distributions of *A*_x_, *A*_y_, *φ*_x_, and *φ*_y_.

Among the seven sub-images, three correspond to the interference patterns between *E*_x_(x, y) and reference fields *R*_m_ = *A*_r_e^i2^*^mπ^*^/3^, where *A*_r_ represents a uniform amplitude, and *m* takes on values 0, 1, and 2. These interference patterns yield intensities that can be expressed as:

$$I_{m}(x,y)=\left|E_{x}(x,y)+A_{r}e^{i2m\pi/3}\right|^{2}$$

(S1)

Consequently, we have

(S2)

(S3)

Then we can obtain:

(S4)

(S5)

The phase of *E*_x_ can be directly obtained as:

(S6)

This equation corresponds to Equation 1 of the main text.

The other four sub-images are the intensities of *E*_x_, *E*_y_, *E*_x_+1i*E*_y_, and *E*_x_+*E*_y_, labeled as$I'_{0}$, $I'_{1}$, $I'_{2}$ and $I'_{3}$, respectively. Therefore, we have

$$I'_{0}=\left|E_{x}\right|^{2}=A_{x}^{2}$$

$$I'_{1}=\left|E_{y}\right|^{2}=A_{y}^{2}$$

Based on the four equations, we can obtain:

(S7)

This corresponds to Equation 2 of the main text. In the actual design, the amplitudes of the two fields *E*_x_ and *E*_y_ are increased by a factor *r*=1.6 to ensure consistency of their peak intensities with the rest. Therefore, the phase difference becomes:

(S8)

**Section 2. Multi-order diffraction design with gradient-based optimization**

In this section, our aim is to design a metasurface capable of producing seven diffraction sub-images of the *E*_x_ and *E*_y_ fields with maximal efficiency. When the input field $E_{i}^{in}(x,y)$ passes through Lens 1, the field at the metasurface plane is represented as:

$$E_{i}^{m}(\eta,\nu)=\frac{\exp(i2kf)}{i\lambda f}\int_{-\infty}^{+\infty} \int_{-\infty}^{+\infty} E_{i}^{in}(x,y)\exp\left[-i2\pi\left(x\frac{\eta}{f\lambda}+y\frac{\nu}{f\lambda}\right)\right]dxdy$$

$\propto F\left[E_{i}^{in}(x,y)\right]$ (S9)

Here, *f* denotes the focal length of both Lenses 1 and 2, *F* represents the Fourier transformation. Assuming the complex-amplitude of the metasurface pattern to be *U*_i_, the light transmitted through Lens 2 and imaged at the focal distance can be defined as:

$$E_{i}^{out}(x,y)=\frac{\exp(i2kf)}{i\lambda f}\int_{-\infty}^{+\infty} \int_{-\infty}^{+\infty} E_{i}^{m}(\eta,\nu)U_{i}(\eta,\nu)\exp\left[-i2\pi\left(\eta\frac{x}{f\lambda}+\nu\frac{y}{f\lambda}\right)\right]d\eta d\nu$$

$$\propto F\left[E_{i}^{m}(\eta,\nu)U_{i}(\eta,\nu)\right]$$

$$\propto F\left\{F\left[E_{i}^{in}(x,y)\right]U_{i}(\eta,\nu)\right\}$$

$=F\left[U_{i}(\eta,\nu)\right]\otimes E_{i}^{in}(-x,-y)$ (S10)

This corresponds to Equation 3 of the main text. To produce seven sub-images with a coefficient of *a*_i_, a straightforward method is:

$U_{i}(\eta,\nu)=\sum_{i} a_{i}\exp(ik_{x}^{i}\eta+ik_{y}^{i}\nu)$ (S11)

termed as the direct plane-wave summation method. This gives:

$$F\left[U_{i}(\eta,\nu)\right]\otimes E_{i}^{in}(-x,-y)$$

$$=\left[\sum_{i} a_{i}\delta(f\lambda k_{x}^{i}/2\pi,f\lambda k_{y}^{i}/2\pi)\right]\otimes E_{i}^{in}(-x,-y)$$

$=a_{i}\sum_{i} E_{i}^{in}(-x+f\lambda k_{x}^{i}/2\pi,-y+f\lambda k_{y}^{i}/2\pi)$ (S12)

This represents a summation of multiple $E_{i}^{in}$ at distinct regions, each with a coefficient *a*_i_. Giving that the center of the sub-images is represented by (*x*_i_, *y*_i_), we can determine:

$$k_{x}^{i}=k_{0}x_{i}/f$$

$$k_{y}^{i}=k_{0}y_{i}/f$$

where *k*_0_=2*π*/*λ*. However, an inherent limitation of this approach is the decreased efficiency with an increasing number of plane waves. Owing to the absence of gain in the metasurface, the maximal amplitude cannot surpass 1.0. After normalizing the metasurface pattern $U_{i}(\eta,\nu)$, the efficiency of the metasurface can be expressed as:

$T=\frac{\sum_{i} \left|a_{i}\right|^{2}}{\left(\sum_{i} \left|a_{i}\right|\right)^{2}}$ (S13)

For our design, we have *a*_1_=*a*_2_=*a*_3_=*a*_6_=*a*_7_=1.0, *a*_4_=1.6, *a*_5_=0.0 for *E*_x_ field and *a*_1_=1.0, *a*_2_=*a*_4_=*a*_6_=*a*_7_=0.0, *a*_3_=1i, *a*_5_=1.6 for *E*_y_ field. The obtained efficiencies are *T*_x_=17.36% and *T*_y_=35.19%.

To address the aforementioned efficiency concerns, we employed gradient-based optimization for designing the metasurface pattern. If we assume the unit period of the metasurface to be *P* and the metasurface pattern exhibits a pure phase of $e^{i\theta_{i}}$ (*i*=*x*, *y*) with a periodicity of *L*=*NP* (where *N*=8) along both *x* and *y* axes, then:

$F\left[U_{i}(\eta,\nu)\right]=\sum_{mn} a_{mn}\delta(x-\frac{mf\lambda}{8P},y-\frac{nf\lambda}{8P})$ (S14)

where $a_{mn}=\frac{1}{L^{2}}\int_{0}^{L} \int_{0}^{L} U_{i}(\eta,\nu)\exp\left[-i2\pi\left(\eta\frac{x}{f\lambda}+\nu\frac{y}{f\lambda}\right)\right]d\eta d\nu=\frac{1}{N^{2}}\text{FFT}(U)$ with FFT symbolizing the fast Fourier transformation.

Subsequently, $E_{i}^{out}(x,y)$ can be expressed as:

$$E_{i}^{out}(x,y)=\sum_{mn} a_{mn}\delta(x-\frac{mf\lambda}{8P},y-\frac{nf\lambda}{8P})\otimes E_{i}^{in}(-x,-y)$$

$=a_{mn}\sum_{mn} E_{i}^{in}(-x-\frac{mf\lambda}{8P},-y-\frac{nf\lambda}{8P})$ (S15)

Here, *a*_mn_ represents for the coefficients of the sub-images. The sub-images of interest are the nearest seven to the zero order, leading to the center-to-center distance between sub-images *D* as *D*=*fλ*/8*P*. In the subsequent discussions, the coefficients of these seven orders *a*_mn_ are denoted as *a*_i_ for brevity.

Our approach aims to enhance the overall efficiency of the seven orders while maintaining consistent coefficient ratios among them. To achieve this goal, our loss function comprises two essential components: efficiency and constraint. As the algorithm is to minimize the loss function, the efficiency term for the *E*_x_ and *E*_y_ fields is expressed as:

$L_{x}=-\left|a_{1}\right|^{2}-\left|a_{2}\right|^{2}-\left|a_{3}\right|^{2}-\left|a_{4}\right|^{2}-\left|a_{6}\right|^{2}-\left|a_{7}\right|^{2}+\left|a_{5}\right|^{2}=-\sum_{i\neq5} \left|a_{i}\right|^{2}+\left|a_{5}\right|^{2}$ (S16)

$L_{y}=-\left|a_{1}\right|^{2}-\left|a_{3}\right|^{2}-\left|a_{5}\right|^{2}+\left|a_{2}\right|^{2}+\left|a_{4}\right|^{2}+\left|a_{6}\right|^{2}+\left|a_{7}\right|^{2}=-\sum_{i=1,3,5} \left|a_{i}\right|^{2}+\sum_{i=2,4,6,7} \left|a_{i}\right|^{2}$ (S17)

where negative signs are associated with existing fields, while positive signs correspond to null intensities.

Furthermore, it is essential to maintain specific ratios between different coefficients, which can be summarized as follows: *a*_1_=*a*_3_, *a*_2_=*a*_6_=*a*_7_, |*a*_1_|=|*a*_2_|, |*a*_4_|=*r*|*a*_2_| for *E*_x_ field, and *a*_3_=1i*a*_1_, |*a*_5_|=*r*|*a*_1_| for *E*_y_ field.

Consequently, the loss due to ratio constraints can be expressed as:

$\sum_{ij} L_{ij}=\left|1-a_{2}/a_{6}\right|^{2}+\left|1-a_{2}/a_{7}\right|^{2}+\left|1-a_{3}/a_{1}\right|^{2}+\left(1-|a_{1}/a_{2}|\right)^{2}+\left(r-|a_{4}/a_{2}|\right)^{2}$ (S18)

$\sum_{ij} L_{ij}=\left|1i-a_{3}/a_{1}\right|^{2}+\left(r-|a_{5}/a_{1}|\right)^{2}$ (S19)

which apply to the *E*_x_ and *E*_y_ fields, respectively. The total loss function combines the efficiency term and the ratio constraints term.

We use PyTorch 1.13 for gradient calculation, which automatically computes gradients. Our training starts with a randomly initialized phase and uses the “Adam” optimizer with an initial learning rate of 3e-2. we fine-tune this learning rate dynamically using 'ReduceLROnPlateau' with a patience of 30, a threshold of 0.01, and a decay rate of 0.9 to ensure efficient model convergence and performance.

Supplementary figure 1 depicts the loss as a function of the iteration. Notably, all constraint terms and the efficiency associated with null fields converge to zero, confirming the preservation of the desired coefficient ratios. The final efficiencies of the *E*_x_ and *E*_y_ fields significantly improve to 80.8% and 80.4%, respectively. A comparison of the efficiency for various orders with direct plane-wave summation and optimization is shown in Figs. S1c-d.

Furthermore, for polarization retrieval, it is necessary that the *a*_1_ coefficients of *E*_x_ field *a*_1x_ and *E*_y_ field *a*_1y_ should be identical. To achieve this, a coefficient *c*=*a*_1x_/*a*_1y_ is multiplied by the metasurface pattern of *U*_y_. In our main text, when we set $U_{x}=0.5e^{i\theta_{x}}$, we have $U_{y}=0.5ce^{i\theta_{y}}=0.39e^{i\theta_{y}}$, where the phase of *c* is incorporated to$e^{i\theta_{y}}$.


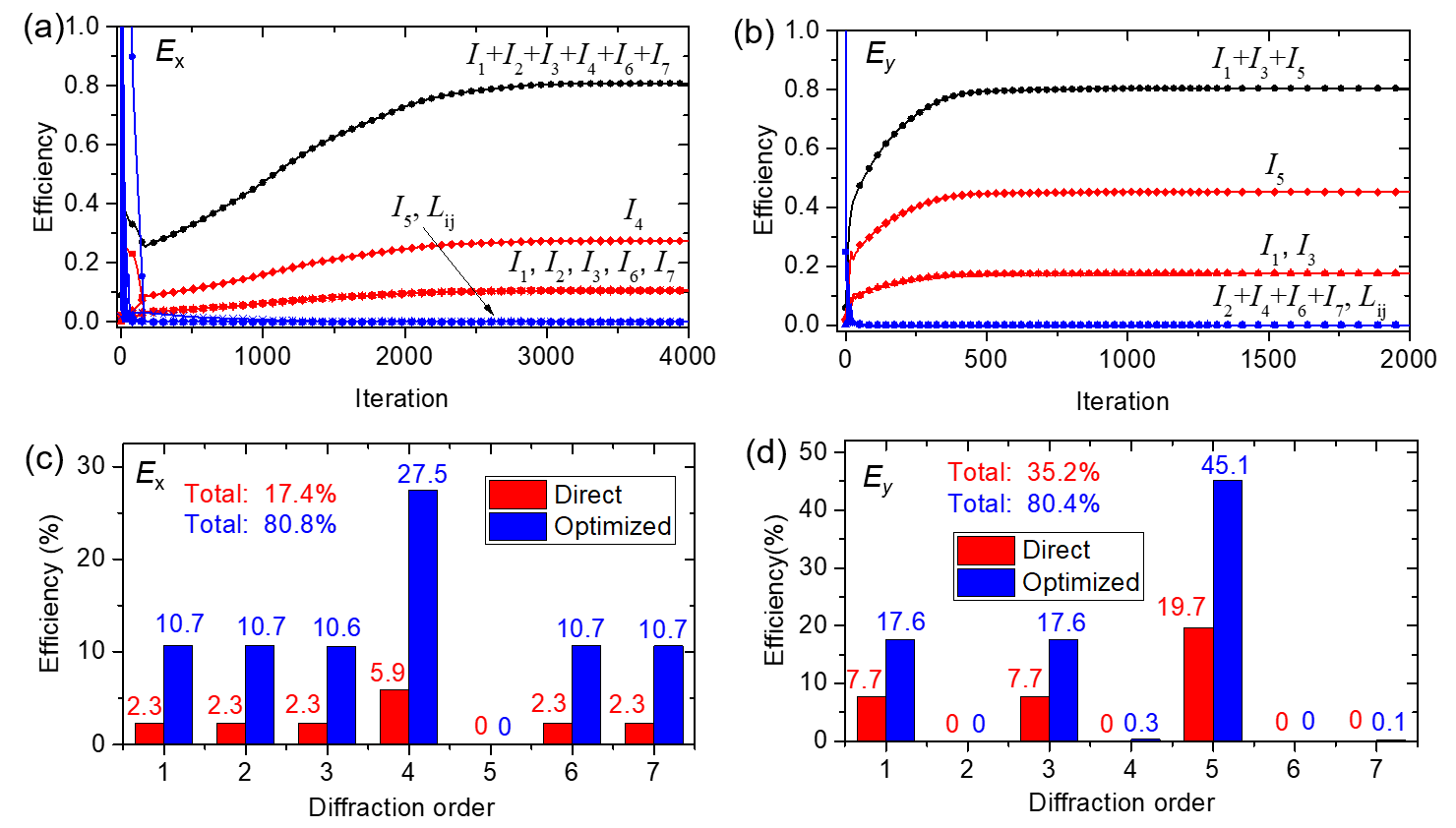


Supplementary Figure 1. Optimization of multi-diffraction orders with constrained Fourier coefficients. (a) Loss value plotted against the number of iterations for *E*_x_ field diffraction. (b) Loss value plotted against the number of iterations for *E*_y_ field diffraction. The black lines represent the total efficiency, while the red line represents the individual efficiency of the order of interest. The blue lines correspond to the order that is intended to be minimized (zeroed). (c, d) Efficiency comparisons for various orders using direct plane-wave summation (red bars) and optimization (blue bars) for both Ex field (c) and Ey field (d).

**Section 3. Optimization of reference field**

The phase retrieval of the *E*_x_ field necessitates the use of three uniform reference fields with equidistant phases in three distinct regions, each having a diameter of *d* (refer to supplementary fig. 2a). As per Eq. S10, the reference field is derived from the input field. The critical question here is how to generate these desired reference fields consistently, regardless of variations in the input field.

Based on the properties of convolution, it becomes evident that if the Fourier transformation of the metasurface pattern exhibits uniform reference fields with e^i2π^*^m^* at three distinct distance regions, each with a diameter of 2*d*, then it will generate the desired reference field within the three circles with a diameter of *d* (supplementary fig. 2b). The generated uniform complex amplitude can be calculated as:

(S20)

Here, we assume that the input field $E_{i}^{in}$ has a field shape with a diameter of *d* and is zero outside. Consequently, the bounds of the integral above are confined to the circular areas. Furthermore, assuming zero field values in the other four circular regions ensures that the generated reference fields remain zero, avoiding interference with other fields. The metasurface pattern can be directly obtained by performing an inverse Fourier transformation of the *F*(*U*) pattern. Assuming zero fields in the regions outside the three circular regions (each with a diameter of 2*d*), the maximum amplitude of the metasurface is calculated to be 719.3.


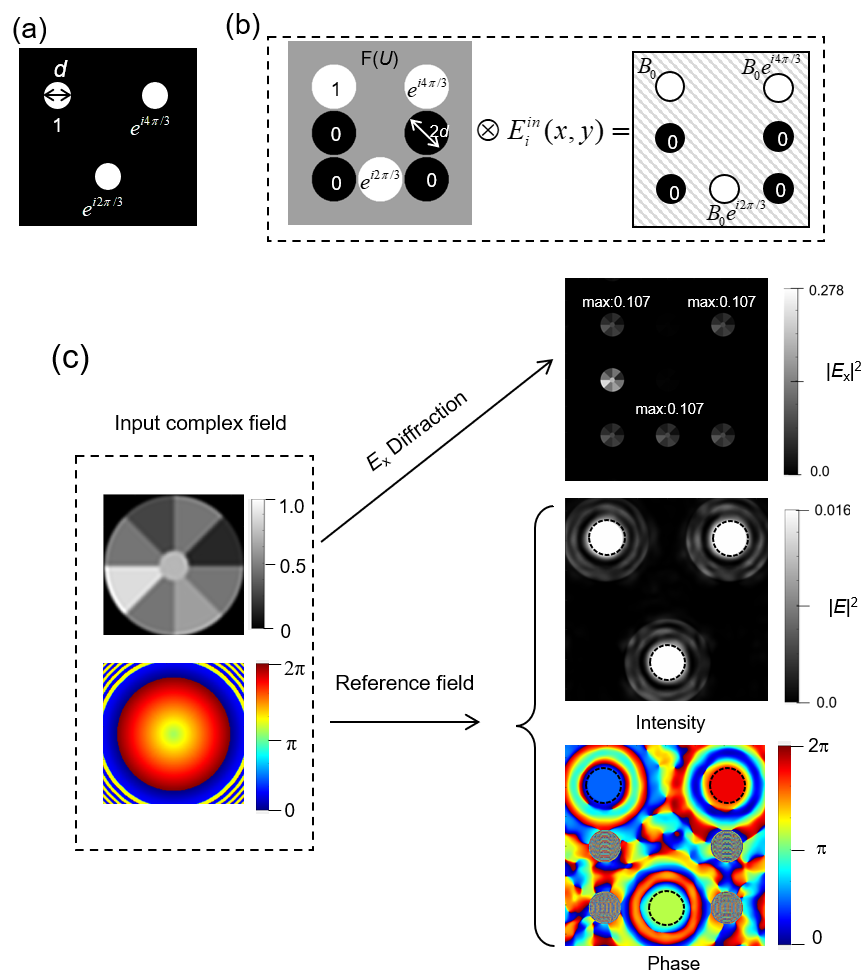


Supplementary Figure 2. Generation of reference field from arbitrary input field. (a) Illustration of an ideal reference field comprising three circular regions, each with a diameter of *d*. (b) The generation of a reference field from an arbitrary input field involves the convolution of the input field with the Fourier transformation of the metasurface pattern *F*(*U*). The uniformity of the reference field is ensured by the uniform *F*(*U*) pattern within seven circular regions, each having a diameter of 2*d.* (c) Generation of the *E*_x_ diffraction and the reference field for an input complex field.

To increase the amplitude of the reference field, we must minimize the maximum amplitude of the metasurface pattern. The complex amplitude in the areas outside the seven circular regions is assumed to be optimized (gray areas in supplementary fig. 2b), and the loss function max(|*U*|) is employed, where *U* represents the complex amplitude of the metasurface. The gradient calculations are performed utilizing PyTorch 1.13, with the parameters consistent with those described in the aforementioned diffraction optimization process. The key distinction lies in the choice of the learning rate, as it significantly influences the maximal amplitude of the metasurface, as shown in supplementary fig. 3. Notably, we have observed that a higher learning rate results in a diminished maximal amplitude of the metasurface. Nevertheless, this also leads to an increase in amplitude in the peripheral regions outside the seven circular areas, consequently giving rise to a larger stray background field. To balance, we have selected a learning rate of 0.06. Post-convergence, the peak amplitude of the metasurface is reduced to 190.1.

A complex field (supplementary fig. 2c) is introduced to examine the reference field and the diffraction field *E*_x_. For a normalized metasurface designed for *E*_x_ field diffraction, it generates seven sub-images with a maximum intensity of 0.278. Among these sub-images, the three interfering with reference field exhibit a maximum intensity of 0.107 (supplementary fig. 2c). In comparison, a normalized metasurface designed for use as a reference field induces a uniform intensity of 0.016 within the three circular regions. Considering all possibilities, the interference between these two fields results in a maximum intensity of $\left|\sqrt{0.107}+\sqrt{0.016}\right|^{2}=0.206$ and a minimal intensity of$\left|\sqrt{0.107}-\sqrt{0.016}\right|^{2}=0.04$, yielding a maximum-to-minimum intensity ratio of 5.14. For the reference field, it can be found that the intensities and phases are uniformly manifested within the delineated three circular regions (denoted by circles of diameter *d*). The phases are in perfect alignment with the design values of 2*mπ*/3. While peripheral stray fields are present around the targeted regions, they do not affect the measurement of the optical fields.


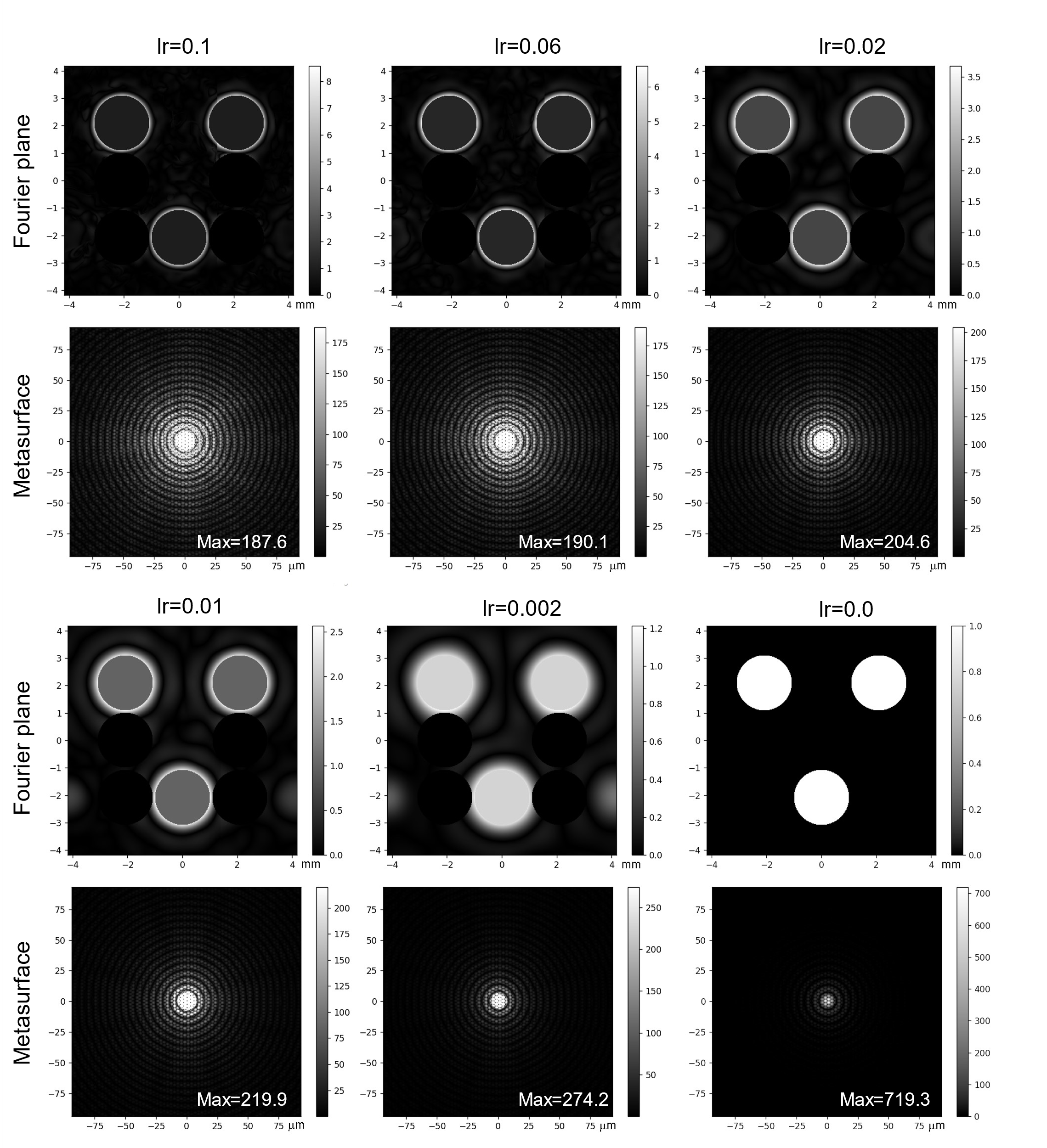


Supplementary Figure 3. The optimized metasurface pattern (bottom) the corresponding Fourier transformation (top) for the generation of reference field with different learning rates. The maximal amplitude of metasurface pattern is indicated in each panel.

It is essential to highlight that the intensity of the reference field is highly dependent on the input field pattern. In certain cases, it may result in significantly lower intensity compared to the *E*_x_ field. To address this issue, we propose the following strategies:

1. Adjusting the linear polarizer axis: Assuming that the linear polarizer is initially set at an angle *θ* (defaulting to 45°), the complex amplitude of the *E*_x_ field for interference $E_{x}^{in}\cos\theta$. In practical design, the reference field is encoded in both *x* and *y* polarizations, and thus, the reference field for interference becomes , where *B* and *φ* represent the amplitude and phase of the uniform reference field. It is evident that increasing the angle *θ* will decrease the *E*_x_ field, resulting in an enhanced contrast of the interference pattern.

2. Modifying the field of view size: In experiments, we have employed an iris to control the size of the input field within a diameter less than *d*=1 mm. Since the complex amplitude of the reference field is obtained by integrating the input field over its area where the existing field is present, the reference field's intensity can be adjusted by modifying the diameter of the iris. Therefore, we can choose an appropriate iris diameter to enhance the reference field intensity.

3. Rotate the metasurface angle. By rotating the metasurface with an angle *θ*, we effectively perform a coordinate transformation of the input field. In the metasurface's coordinate system, the input *E*_x_ and *E*_y_ fields become $E_{x}^{in}\cos\theta+E_{y}^{in}\sin\theta$ and $-E_{x}^{in}\sin\theta+E_{y}^{in}\cos\theta$ , respectively. This rotation will indeed impact the *E*_x_ field and can be utilized to decrease the maximal of it, thereby enhancing the contrast for interference.

**Section 4. Jones matrix metasurface design with nanoblock elements**

The Jones matrix of metasurface is designed with $J_{xx}=0.5e^{i\theta_{x}}+0.5U_{norm}$ and $J_{yy}=0.39e^{i\theta_{y}}+0.61U_{norm}$, where the maximal amplitudes are both normalized to unity. To realize this specific Jones matrix, we employ a unit cell composed of two distinct rectangular nanoblocks labeled as *A* and *B*. The transmission coefficients through the *x* and *y* axes are denoted as and for *A*, and and for *B*, respectively. The total Jones matrix of the unit cell can be expressed as:

(S21)

(S22)

Here, the factor 1/2 is a normalized coefficient for the Jones matrix. The four phase terms , , and can be directly obtained as:

(S23)

(S24)

(S25)

(S26)

For the nanoblock structure, it supports two propagation modes along its *x* and *y* axes. The transmission magnitude and phase shift with *x*-polarized and *y*-polarized incidences, as functions of the transverse dimensions of the nanoblocks dx and dy are illustrated in supplementary fig. 4. The period is 350 nm, the height of the nanoblock is 600 nm and the wavelength is 780 nm. To achieve a desired phase retardation of *φ*_x_ and *φ*_y_, the transverse dimensions of the nanoblocks are selected according to the following steps:

(1) Begin by setting a pre-defined average transmission magnitude $t_{avg}$. This value is primarily determined by the overall transmissions of the nanoblocks with different transverse dimensions and should not be too small. In our work, we have chosen $t_{avg}=0.98$.

(2) Calculate the complex-valued errorsand  , and choose the large one $\varepsilon_{\max}=\max(\varepsilon_{x},\varepsilon_{y})$.

(3) Determine the configuration that minimizes $\varepsilon_{\max}$ for all possible dimensions.

These steps help in obtaining the desired phase shifts by selecting appropriate transverse dimensions for the nanoblocks while maintaining an acceptable transmission magnitude.


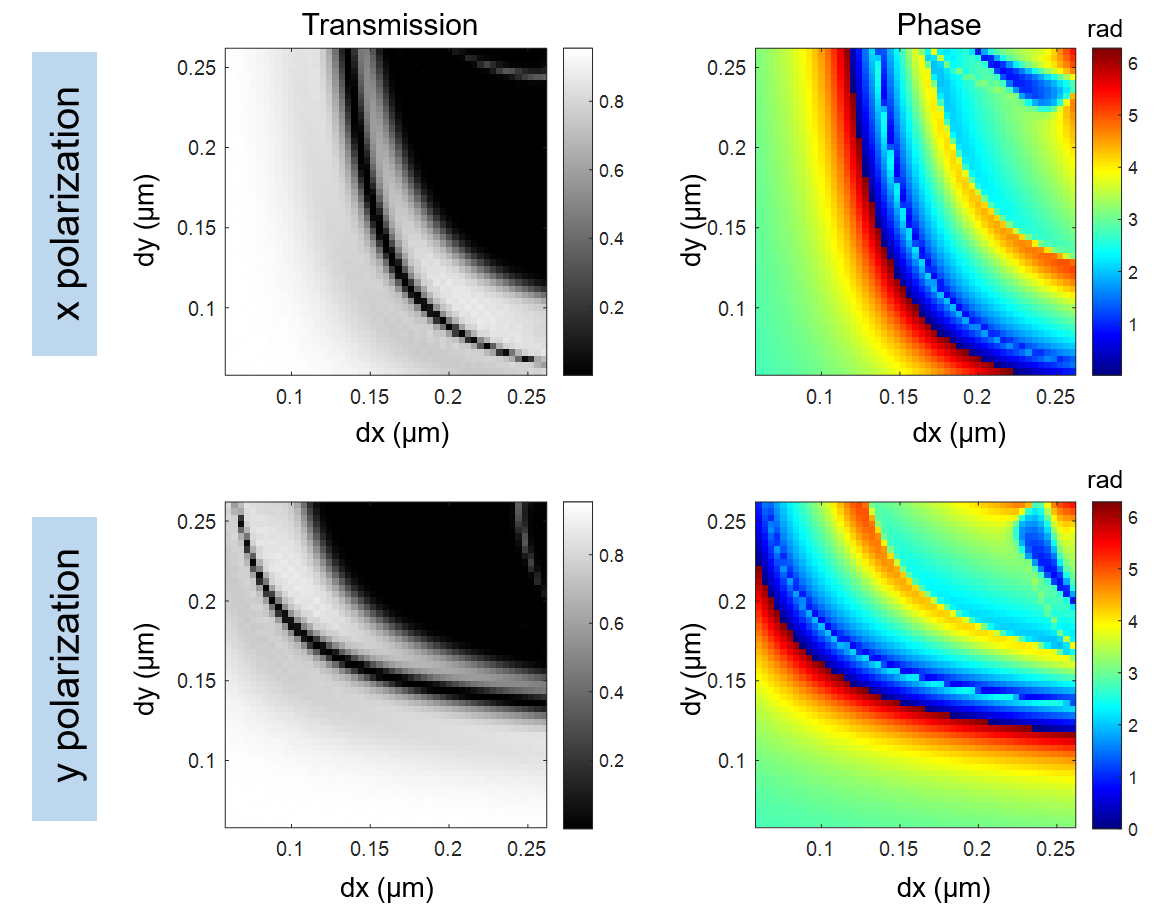


Supplementary Figure 4. Metasurface unit design. Transmission and phase shift (radians) of the nanoblock as a function of the transverse dimensions, dx and dy, for *x*-polarized and y-polarized incidences. The optical response for *y*-polarization response is obtained by swapping *x* and *y* of x-polarization response due to the nature of symmetry.

**Section 5. Encoding arbitrary complex-amplitude distribution with pure-phase SLM**

In this section, we aim to use the Spatial Light Modulator (SLM) to impose arbitrary amplitude and phase distributions with pure phase modulation. The SLM used in our setup is the PLUTO-2-NIR-011 from HOLOEYE, which is a phase-only SLM with a pixel pitch of *P*=8 μm. At the designed wavelength of 780 nm, the SLM enables an effective diffraction numerical aperture (N.A. sin*θ*) of λ/2*P*=0.0488 with the center located at zero.

On the other hand, the designed metasurface has a diameter of 600 μm, and the focal length of the lens in the 4*f* system, as shown in Fig. 2a, is 15 mm, indicating an N.A. of 0.02. It might appear that the diffraction field generated by the SLM can cover the required N.A. for the metasurface. However, this is not the case. When using pure phase modulation to generate a complex amplitude, the N.A. of this complex-amplitude field is inherently limited to a very small value, much smaller than the N.A. of the pure phase distribution.

To overcome this limitation, we employ two lenses to scale the SLM patterns. In Fig. 3c, the focal lengths of lenses 1 and 2 are chosen as 150 mm and 50 mm, respectively. As a result, the generated arbitrary field has a period of 8/3=2.67 μm and the effective diffraction N.A. is increased to 0.14625. With such an N.A. of pure phase, we can generate arbitrary complex amplitudes within an N.A. of 0.02.

Assuming that the generated arbitrary complex amplitude is represented as *U*(*x*,*y*)=*A*(*x*,*y*)exp(1i*φ*(*x*,*y*)), with a normalized *A* and the center spatial spectrum located at zero, the pixel number is set to 360×360, ensuring its size remains less than 1 mm. We have chosen three different optical field configurations, as shown in the first column in supplementary fig. 5. The phase distribution for the three types is as follows:

For *E*_x_ field (type 1):

(where θ is the azimuthal angle) (S27)

For *E*_y_ field (type 1):

(where r is the azimuth radius) (S28)

For *E*_x_ field (type 2):

(S29)

For *E*_y_ field (type 2):

(S30)

For *E*_x_ field (type 3):

(S31)

For *E*_y_ field (type 3):

(S32)

Firstly, we limit the N.A. of these fields to 0.02 using a Gaussian filter and spectrum truncation. The results are shown in the second column in supplementary fig. 5, which exhibit no significant difference from the original fields but with a slight blur.

Subsequently, we employ type 3 of the pure phase holograms [[^1^](#_ENREF_1)] to generate the desired complex amplitude. In detail, we first add a phase term -*kδx* to *φ*(*x*,*y*), i.e., *φ*’(*x*,*y*)= *φ*(*x*,*y*) -*kδx* , where *k*=2*π*/*λ* and *δ*=0.123. This adjustment shifts the center of the N.A. of *U*(*x*,*y*) to (-*δ*, 0). We can then obtain the pure phase of the SLM, denoted as Ψ=inversebessel1(0.5819*A*)sin(*φ*’), where inversebessel1 is the inverse of the first-order Bessel function. This pure phase distribution generates the desired complex field with an N.A. of 0.02 at center of (-*δ*, 0). We subsequently shift the center to zero by adding a phase term of *kδx*, i.e., $\exp(ik\delta x)\cdot\exp(i\Psi)$, with the phase shown in the third column of supplementary fig. 5. With this pure phase distribution, the obtained amplitude and phase with limited N.A. of 0.02 (fourth column in supplementary fig. 5) closely align with our design.

Finally, we apply this phase to the SLM. It's important to note that a gradient phase term needs to be added to compensate for the gradient phase introduced by oblique incidence. The fifth column in supplementary fig. 5 presents the measured intensity of the field generated using the pure phase on the SLM. It closely aligns with our design, albeit with some speckle noises present. When the metasurface is positioned at the center of the Fourier plane, the generated sub-images exhibit similar speckle noise patterns. This observation suggests that the metasurface has effectively diffracted the generated images into multiple orders, and the speckle noise is not a consequence of the metasurface itself. The obtained images with (Fig. 4 in the main text) and without the metasurface (fifth column in supplementary fig. 5) are compared, with structural similarity (SSIM) values around 0.82.


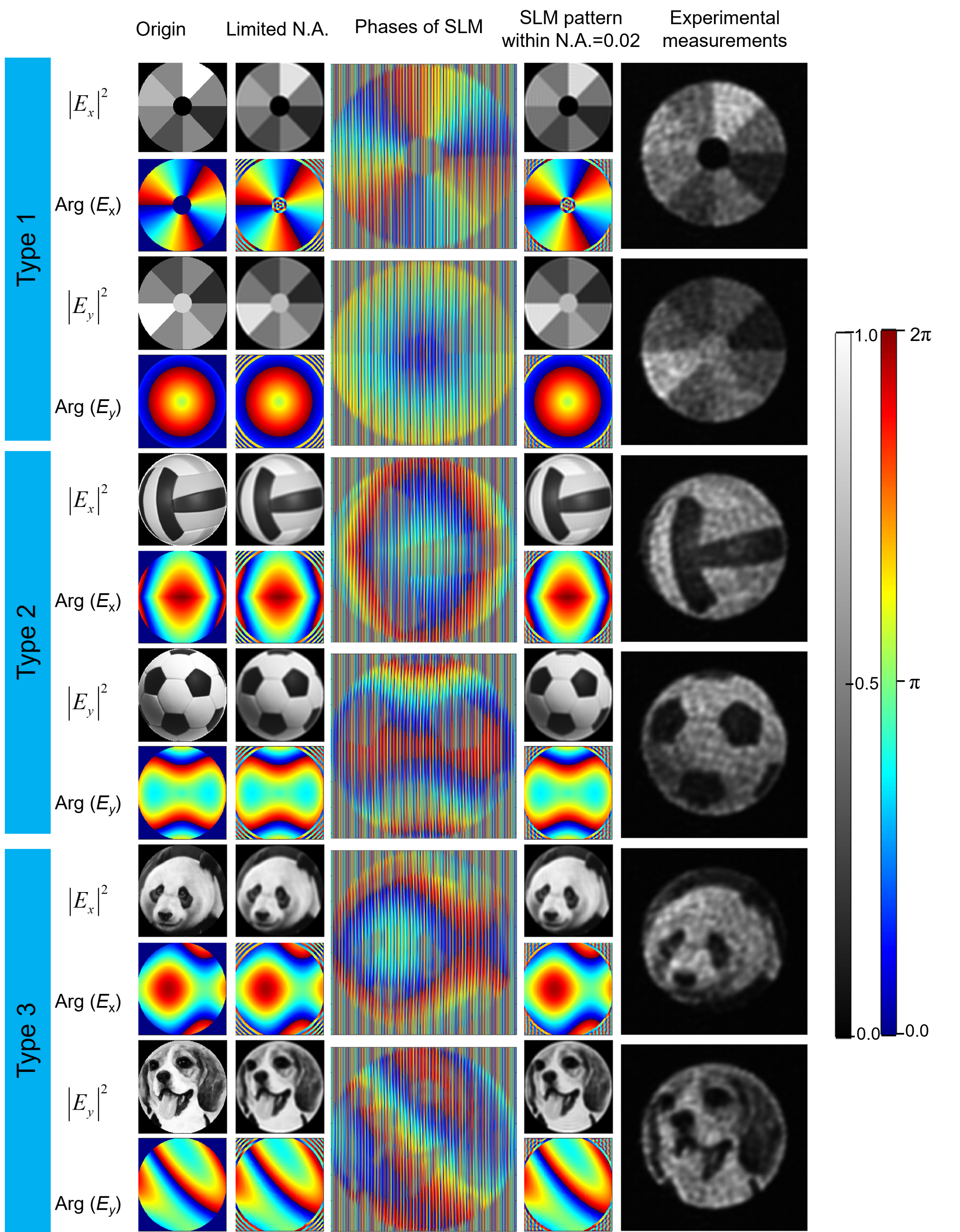


Supplementary Figure 5. Encoding three types of complex-amplitude distribution with pure-phase modulation. The figure consists of five columns. First column displays the original amplitude and phase distributions. Second column depicts the amplitude and phase distributions limited by N.A.=0.02 through the use of a Gaussian filter and spectrum truncation. Third column shows the pure phase distribution employed to generate the corresponding complex amplitude. Fourth column illustrates the complex amplitude resulting from the pure phase distribution within N.A.=0.02. Fifth column shows the experimental measurement of intensity distribution with pure phase imposed on the SLM.

**Section 6. Lens choose and image correction**

Recalling the optical system based on the metasurface (Figure 2b of main text), it consists of two lenses and a metasurface. The input field is positioned at the center of the front focal plane of Lens 1 and is focused at the center of the back focal plane. Consequently, for Lens 1, a conventional spherical lens is a suitable choice, as it introduces minimal spherical aberration. However, as the field passes through the metasurface, it undergoes diffraction into several orders with large diffraction angles and is subsequently imaged at regions located at a certain distance from the center. This results in significant spherical aberration. To mitigate this spherical aberration, we select an aspheric lens (AL1815, Thorlabs, Inc.) for Lens 2.

We conduct a ray tracing simulation to compare two scenarios: one with a conventional spherical lens and the other with an aspheric lens for Lens 2. The results are presented in supplementary fig. 6. In this simulation, a grating is placed at the center Fourier plane to diffract the incident field with sin(*θ*_x_)=2/15 and sin(*θ*_y_)=-2/15, corresponding to the one at the corner of the seven sub-images. It can be observed that the aspheric lens can achieve better focus with clearer imaging compared to the spherical lens, thereby reducing spherical aberration. However, both cases exhibit some degree of image distortion due to the large diffraction angle.


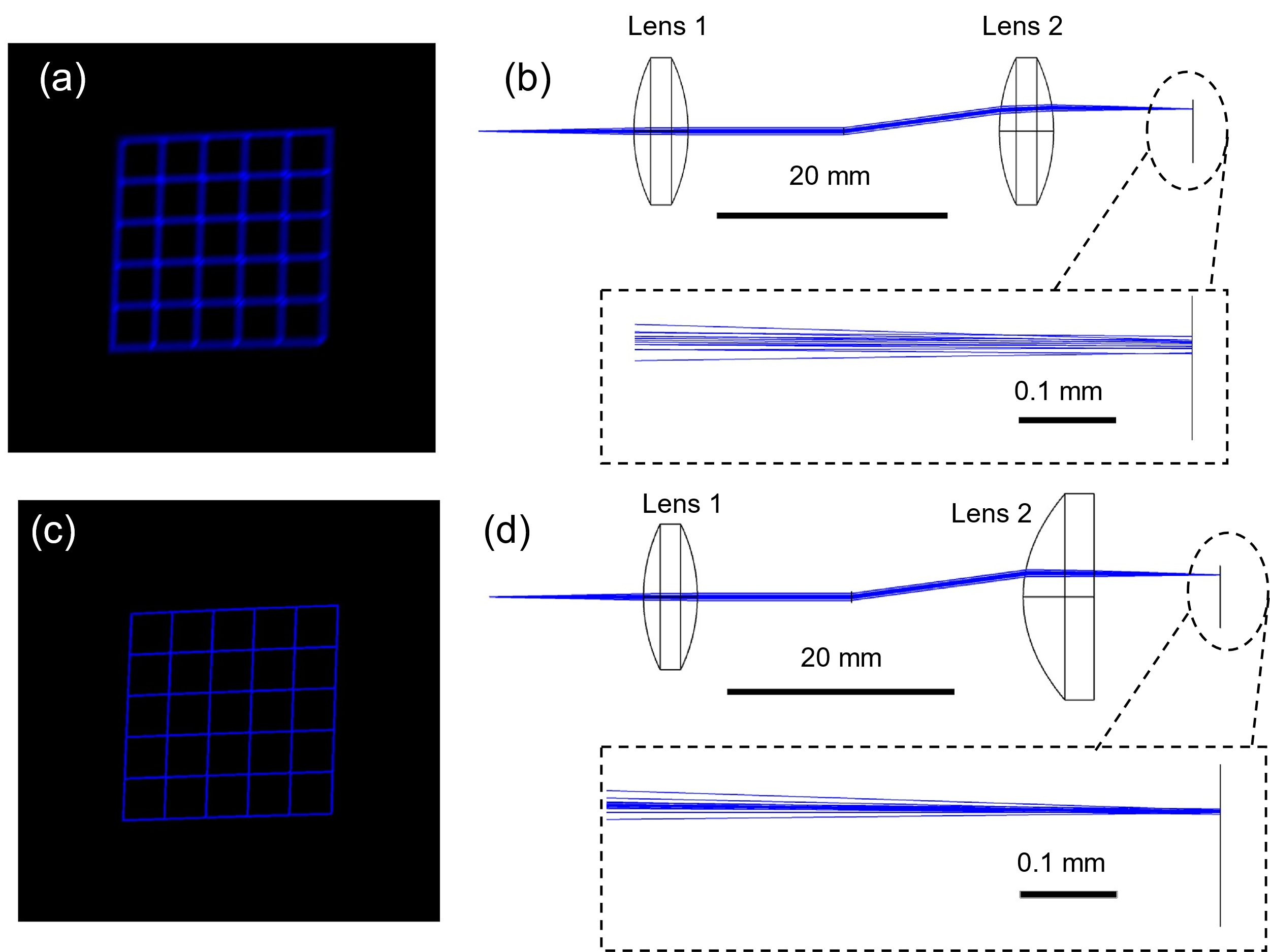


Supplementary Figure 6. Ray tracing simulation comparison between two different types of lenses. (a-b) Image simulation of a grid pattern (a) and point-to-point imaging (b) with a spherical lens for Lens 2. (c-d) Image simulation of a grid pattern (c) and point-to-point imaging (d) with an aspheric lens for Lens 2. The enlarged insets in (b) and (d) provide a closer look, highlighting the superior focusing capabilities of the optical system employing the aspheric lens.

To correct this distortion, a checkerboard image (supplementary fig. 7a) is employed as the input, and its focused images of the seven diffraction orders are simulated by implementing gratings with different diffraction angles. The results are shown in supplementary fig. 7b, where various levels of distortion can be observed in all seven images. In our experiment, we observe similar distortions, as demonstrated in the center column of Figure 4 in the main text.

Subsequently, the pattern points of the checkerboard are detected and labeled with circles in supplementary figs. 7a-b. The points in supplementary fig. 7a serve as the base control points, and the points in the seven sub-images (supplementary fig. 7b) are used as input points to be transformed. We utilize the "cp2tform" function in MATLAB to compute a transformation that best fits the mapping from the input points to the base points, employing a polynomial transformation type, which is referred to as "tform". This transformation is then applied (using the "imtransform" function in MATLAB) to the seven distorted images, resulting in the corrected images (supplementary fig. 7c). This transformation is applied to our measured results for image correction, resulting in the full field distribution displayed in the rightmost column of Fig. 4 of main text.


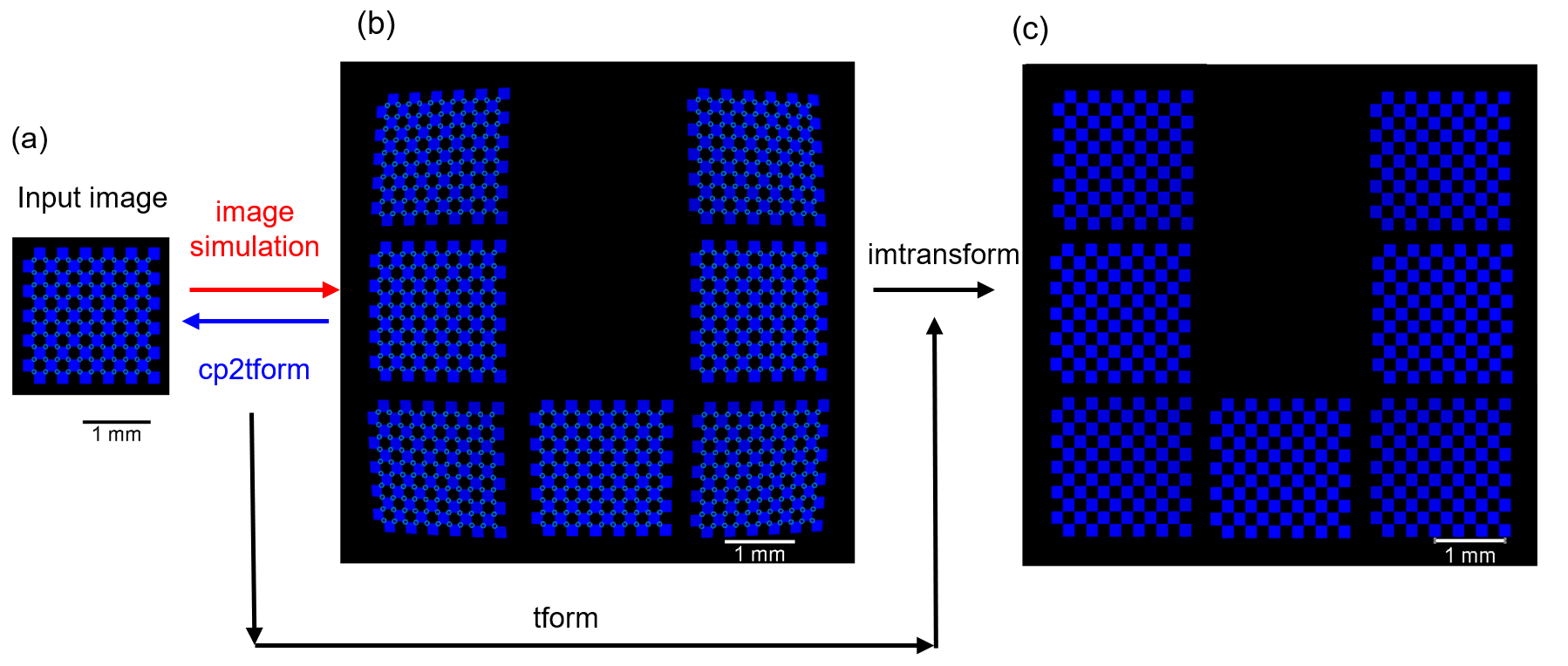


Supplementary Figure 7. Imaging correction. (a) Input checkerboard pattern. (b) Simulated images with ray tracing for the seven diffraction orders. The pattern points in (a) and (b) are marked with circles and are used as reference control points and input points for transformation correction. The correction is achieved using the “cp2tform” function in Matlab. (c) The transformed images of the seven orders with the “tform” obtained from the previous transformation.

**Section 7. Metasurface efficiency measurement**

To measure the efficiency of the metasurface for imaging, we initially projected the designed optical patterns onto SLM and removed the metasurface. In this configuration, the measured optical image in the CMOS camera consists of a single image, with its power measured as *I*_in_. Then, we placed the metasurface at the center Fourier plane of the 4*f* system and captured seven sub-images and one zero-order image in the CMOS camera. The power of the seven images of interest is measured as *I*_out_. The efficiency is defined as *I*_out_ / *I*_in_.

It's essential to note that this efficiency is not constant but depends on the input field. This is because the incident field is the Fourier transform of the input field. When the input field changes, the incident field on the metasurface changes as well, resulting in a change in the transmitted light through the metasurface. For the three specific optical fields presented in Figure 4 of the main text, the measured efficiency falls between 17.5% to 22.4%.

References

[1] V. Arrizón, U. Ruiz, R. Carrada, L. A. González, *JOSA A* **2007**, *24*, 3500.
